# Assessment of jaw bone mineral density, resorption rates, and oral health in patients with severe hemophilia: a case-control study

**DOI:** 10.2340/aos.v83.40337

**Published:** 2024-04-10

**Authors:** Gülin Acar, Alper Aktaş

**Affiliations:** Oral and Maxillofacial Surgery, Hacettepe University, Ankara, Turkey

**Keywords:** Alveolar resorption, bone mineral density, gingival indexes, hemophilia, oral hygiene

## Abstract

**Objective:**

Knowledge about oral hygiene, gingival bleeding, mineral density, and resorption of jaw bones in patients with hemophilia is limited. We evaluated the periodontal and bone status in such patients.

**Material and methods:**

Forty-eight patients with severe type A/B hemophilia and 49 age- and sex-matched controls were included. Assessments included simplified oral hygiene index (OHI-S), calculus index, debris index, gingival index (GI), gingival bleeding time index (GBTI), and decayed, missing, and filled teeth index (DMFTI). Bone resorption was evaluated using panoramic mandibular index (PMI), mental index (MI), and alveolar crest ratio (ACR). Mineral density in the condyle, angulus, and premolar areas was assessed using fractal analysis, with fractal dimensions denoted as condyle fractal dimension (CFD) for the condyle, angulus fractal dimension (AFD) for angulus, and premolar fractal dimension (PFD) for premolar region.

**Results:**

The mean scores were DMFTI = 11.77, OHI-S = 2.44, PMI = 0.268, MI = 5.822, GI = 3.02, GBTI = 2.64, ACR = 2.06, CFD = 1.31, AFD = 1.31, and PFD = 1.17 in the hemophilia group and DMFTI = 11.449, PMI = 0.494, MI = 7.43, GI = 0.67, GBTI = 0.98, OHI-S = 1.45, ACR = 2.87, CFD = 1.35, AFD = 1.35, and PDF = 1.23 in the control group. Differences were significant for all parameters (*p* < 0.005) except for the DMFTI index.

**Conclusions:**

Because of poor oral hygiene, high bone resorption, and low bone mineral density in these patients, clinicians should consider potential bone changes when planning to treat these patients.

## Introduction

Bleeding disorders are life-threatening diseases that humans have faced since ancient times, and the number of diagnosed patients increases every year. Hemophilia is a type of genetically inherited bleeding with several subgroups. In patients with hemophilia A, the most common type, the level of clotting factor 8 in the blood is insufficient, whereas in those with hemophilia B, the level of factor 9 is insufficient. The clinical severity of the disease is inversely proportional to blood factor levels. In the most severe type, the blood factor level is <4% [1].

Owing to the high risk of life-threatening bleeding in patients with hemophilia, many precautions are needed in the field of dentistry. Because 14% of all patients with hemophilia are diagnosed for the first time because of severe oral hemorrhage, dentists should be cautious [2]. Bleeding episodes in patients with hemophilia may occur spontaneously, and inadequate oral hygiene may worsen the situation.

Over the years, the life expectancy of patients with hemophilia has been increasing owing to the development of improved treatments, with their average life expectancy being similar to that of the healthy population. This increase in life expectancy has paved the way for the identification of comorbid diseases associated with hemophilia. In particular, decreased bone density and increased fracture risk in patients with hemophilia have been the areas of focus in recent studies [3]. For instance, to explore bone mineral density in patients with severe hemophilia A, Wallny et al. conducted a study in 2007 where they screened 62 patients for whole-body bone density. Their findings revealed that 16 patients (25.8%) were diagnosed with osteoporosis, and 27 patients (43.5%) met the criteria for osteopenia [4]. In a more recent study conducted in 2020, Ehsanbakhsh et al. aimed to investigate the prevalence of decreased bone density and its associated factors in individuals with hemophilia. Specifically, they compared 57 patients with hemophilia to 60 healthy individuals, demonstrating statistically significant lower lumbar bone density in the patients with hemophilia group. Age, body mass index (BMI), percentage of hemophilia, vitamin D, hepatitis, and smoking were investigated as related factors, and only BMI was found to be correlated with bone density [5]. A case-control study investigating bone mineral density in patients with hemophilia B reported that bone density decreased in the lumbar and femur regions in the severe hemophilia B group compared to that in the control group [6], and concluded that the decreased bone density was similar to that of patients with hemophilia A [6, 7]. The literature has increasingly documented reduced bone density and increased fracture risk in patients with hemophilia. Initially, this was attributed to reduced physical activity, hemarthrosis resulting in arthropathy, acquired immunodeficiency virus, or blood-borne infections such as hepatitis C [8]. However, in a study conducted in an animal experimental model in 2021 [3], which examined the effects of deficient clotting factors in hemophilia on osteoblasts and osteoclasts, it was shown that deficient or defective FVIII or FIX disrupted FX, causing insufficient or defective thrombin formation. In light of this information, in vitro studies showed that thrombin induces osteoblast differentiation and negatively regulates osteoclast formation and function [9]. Some researchers suggest that factor deficiency in hemophilia might directly or indirectly impact the RANK/RANKL/OPG axis and/or the Wnt/β pathway, potentially through the modulation of cytokines and other related factors [7].

However, as the mineralization and resorption patterns of the jaw bones are different from those of other bones, large-scale studies focusing on the jaw bones are needed. Notably, few studies have examined the status of jaw bones in the existing literature. To the best of our knowledge, the sole study in the literature that delved into jaw bone resorption in individuals with hemophilia revealed a significant correlation between panoramic radiographic markers and markers related to bone turnover in patients with hemophilia [10]. Given the limited number of these studies, further research on this subject is warranted.

In addition, with the increase in quality of life, it is necessary to determine the oral hygiene and gingival bleeding patterns of patients with hemophilia with the most appropriate method and to apply an appropriate treatment. Therefore, more studies focusing on oral hygiene and gingival bleeding patterns of patients with hemophilia are needed.

In this study, we aimed to determine the oral hygiene and bleeding status of patients with severe hemophilia using different indices. The relationships among hemophilia, oral hygiene, and bleeding were also investigated. The mineral densities and resorption of the jaw bones of the patients were measured and compared with those of the control group.

## Materials and methods

The study included 48 patients diagnosed with severe (*F* < 4%) hemophilia A or B who agreed to participate in the study, had no additional systemic health problems, and did not smoke or have an intraoral prosthesis. A control group of 49 individuals with the same characteristics but without systemic diseases was also included. In the study group, patients were carefully selected to ensure that they had no diseases other than hemophilia, particularly those affecting bone metabolism, and were not taking any medications. The control group consisted of individuals with no prior health issues or drug usage that could impact bone metabolism. Patients underwent an examination for temporomandibular disease (TMD), and those who exhibited clinical or radiographic evidence of TMD or were suspected of having bruxism were excluded from the study. Furthermore, the medical records of the patients were carefully reviewed, and individuals with vitamin or mineral deficiencies, such as calcium, vitamin D, or iron, which could potentially impact bone metabolism, were excluded from the study. BMI was computed by dividing the body weight (in kilograms) by the square of the height (in meters), and individuals with a BMI falling below 18 (underweight) or exceeding 25 (overweight) [5] were not included in the study. All the radiographic and clinical measurements were performed by a dentist with 6 years of experience who was in the fourth year of specialized training. Panoramic images were obtained using Veraview IC5 HD (Morita Corporation, Osaka, Japan) with the following settings: 12 mA, 18 s, and 70 kV.

The simplified oral hygiene index (OHI-S), an indicator of oral and dental health, was determined using the simplified calculus index (CI-S) and debris index (DI-S) [11]. The CI-S and DI-S scores are listed in Table 1. The OHI-S of each individual was determined by the mathematical summation of the CI-S and DI-S.

**Table 1:** Debris index (DI-S), calculus index (CI-S) [11], and gingival index [12].

	DI-S	CI-S	Gingival index
**0**	No debris or stain	No calculus	Normal gingiva
**1**	Soft debris covering not more than one-third	Supragingival calculus covering not more than one-third	Mild inflammation, slight color change, and edema
**2**	Soft debris covering more than one-third but not more than two-thirds	Supragingival calculus covering more than one-third but not more than two-thirds	Moderate inflammation, redness, edema and glazing, bleeding on probing
**3**	Soft debris covering more than two-thirds	Supragingival calculus covering more than two-thirds	Severe inflammation, marked redness and edema, ulceration, spontaneous bleeding

DI-S, debris index; CI-S, calculus index.

The degree of gingival inflammation was evaluated using the gingival index (GI) [12] (Table 1) and gingival bleeding time index (GBTI) [13]. The number of caries and filled and missing permanent teeth was determined using the decayed, missing, and filled teeth index (DMFTI).

For CI-S, DI-S, GI, and GBTI scoring, the teeth were evaluated on four surfaces; the sum of the surface scores was divided by four, and the simple average obtained was used as the tooth score. Individual scores were determined by adding the scores of the teeth and dividing them by the number of teeth. The following tooth numbers were used for measurements: 16, 26, 11, 31, 36, and 46.

**Figure 1:**
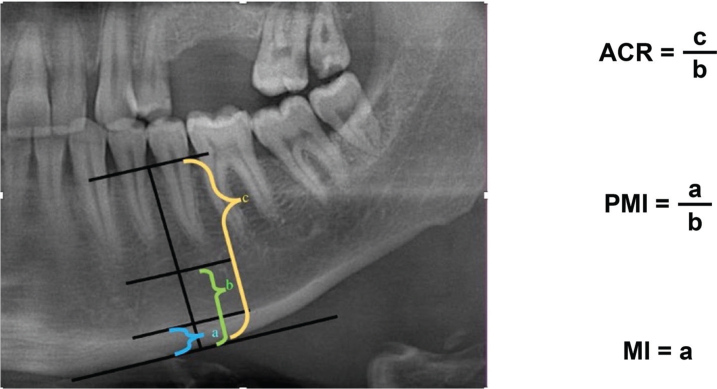
(Left) Line perpendicular to the line parallel to the mandibular cortex, passing through the center of the mental foramen, which was used for evaluation (Right). Calculations of mathematical ratios. ACR: alveolar crest ratio, PMI: panoramic mandibular index, MI: mental index.

Bone resorption and mineral density were evaluated using panoramic radiographs. The amount of resorption in the jaw bones was evaluated using the mental index (MI), panoramic mandibular index (PMI) [14], and alveolar crest ratio (ACR) [15] using a two-point measurement method. The MI, PMI, and ACR values are shown in Figure 1. Two-point measurements were performed using a widely available imaging program (https://download.slicer.org/).

**Figure 2:**
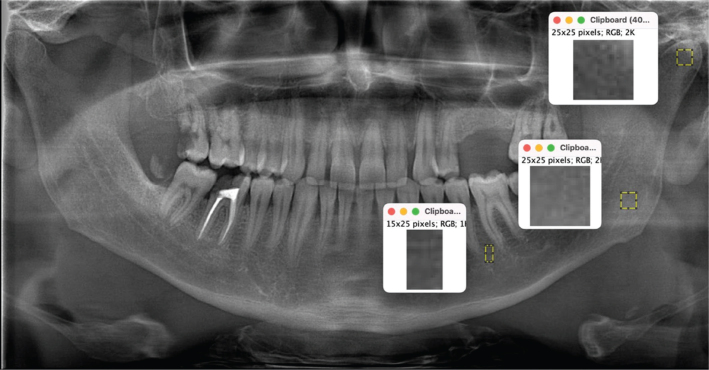
Regions of interest on the condyle, angulus, and premolar regions for fractal analysis.

The mineral densities of the jaw bones were analyzed using fractal analysis (FA) values based on the scans of the angulus, premolar, and condylar regions. FA was performed using the method described by White and Rudolph [16], that is, the morphological properties of the trabecular bone were recorded. The scans used for FA and subsequent fractal dimension (FD) values are shown in Figure 2. For FA, six regions of interest (ROIs) were determined, three each on the condyle, angulus, and premolar regions, symmetrized on the right and left (Figure 2). When determining the ROIs, care was taken not to include anatomical structures, such as the lamina dura, periodontal ligament space, and mandibular nerve. Considering the effect of bacterial plaque on the alveolar crest, it was positioned as apically as possible. The ROIs in the condylar and angulus region covered a size of 25 × 25 pixels, and the ROIs in the premolar region covered a size of 25 × 15 pixels. The numerical values obtained for the right and left sides were averaged. FA was performed using a publicly available imaging program (ImageJ, 1.52p; National Institutes of Health, Bethesda, MD, USA; https://imagej.nih.gov/ij/download.html).

SPSS Statistics version 23 (IBM Corp., Armonk, NY, USA) was used for statistical analysis of the data. The correlations between categorical variables were analyzed using the Pearson’s chi-square test. The compliance of the numerical data to normal distribution was evaluated using visual and analytical methods (Kolmogorov–Smirnov and Shapiro–Wilk normality tests). The significance test of the difference between two means (Student’s t-test/independent samples *t*-test) was used when the parametric test assumptions were met, and the Mann–Whitney *U* test was used when they were not. Significance was set at *p* < 0.05. To calculate the effect size of the study, Cohen’s d coefficient was used when parametric test assumptions were met, and the effect size (*r*) coefficient was used when parametric test assumptions were not met. The measurements were carried out in a computerized environment and were selected randomly from the cohort of patients included in the study. Measurement reliability was evaluated using the intraclass correlation coefficient (ICC). Twenty-five patients, constituting 25% of the dataset, were randomly chosen from the group of 97 patients in the study to identify any potential methodological error, and the same investigator repeated these measurements 2 weeks after the initial assessments. Consequently, the measurements demonstrated a high degree of consistency and reliability.

## Results

The participants’ age, sex, type of hemophilia, and severity of hemophilia, and the results of the statistical analyses are summarized in Table 2.

**Table 2:** Results of statistical analyses.

	Hemophilia (*n* = 48)	Control (*n* = 49)	*p*	Effect size
Female	10	12	0.067	
Male	38	37		
Average age (years)	370.56	42.04	0.077	
Hemophilia A	38			
Hemophilia B	10			
	Mean	Mean		
DMFTI	11.7708	11.449	0.778	0.54
PMI	0.268	0.494	0.001*	2.844
MI	5.822	7.438	0.001*	1.372
GI	3.02	0.67	<0.001*	0.706
GBTI	2.64	0.98	<0.001*	0.764
OHI-S	2.44	1.45	0.001*	0.337
CI-S	1.15	0.67	0.004*	0.294
DI-S	1.3	0.78	<0.001*	0.331
ACR	2.06	2.87	<0.001*	0.395
CFD	1.31	1.35	<0.001*	0.593
AFD	1.31	1.35	<0.001*	0.615
PFD	1.17	1.23	<0.001*	0.639

* *p* < 0.005.

DMFTI: decayed, missing, and filled teeth index, PMI: panoramic mandibular index, MI: mental index, GI: gingival index, GBTI: gingival bleeding time index, OHI-S: simplified oral hygiene index, CI-S: calculus index, DI-S: debris index, ACR: alveolar crest ratio, CFD: condyle fractal dimension; AFD: angulus fractal dimension, PFD: premolar fractal dimension

Correlations among the CI-S, DI-S, OHI-S, GI, and DI-S were analyzed to evaluate the effect of oral hygiene status on intraoral inflammation and bleeding (Table 3). To evaluate the relationship between the amount of bone resorption and mineral density, the correlations of the PMI, MI, and ACR of the patients with the condyle fractal dimension (CFD), angulus fractal dimension (AFD), and premolar fractal dimension (PFD) values were analyzed (Table 4).

**Table 3:** Correlation values of patients with hemophilia and the control group.

			GBTI	OHI-S	GI	DMFTI
OHI-S	Hemophilia	CC	0.018	1	0.362	0.383
		p	0.906		0.011	0.007
	Control	CC	0.611	1	0.630	0.028
		p	0		<0.001	0.849
GI	Hemophilia	CC	0.165	0.362	1	0.197
		p	0.262	0.011		0.18
	Control	CC	0.813	0.630	1	0.187
		p	<0.001	<0.001		0.198
DMFTI	Hemophilia	CC	−0.033	0.383	0,197	1
		p	0.822	0.007	0.18	
	Control	CC	0.123	0.028	0.187	1
		p	0.398	0.849	0.198	

CC: correlation coefficient, OHI-S: simplified oral hygiene index, GI: gingival index, DMFTI: decayed, missing, and filled teeth index, GBTI: gingival bleeding time index

**Table 4:** Correlation values of all individuals.

		CFD	AFD	PFD
ACR	CC	0.514	0.473	0.527
	p	<0.001	<0.001	<0.001
PMI	CC	0.512	0.522	0.554
	p	<0.001	<0.001	<0.001
MI	CC	0.347	0.366	0.338
	p	<0.001	<0.001	0.001

CC: correlation coefficient, ACR: alveolar crest ratio, PMI: panoramic mandibular index, MI: mental index, CFD: condyle fractal dimension, AFD: angulus fractal dimension, PFD: premolar fractal dimension

No significant difference was found between the patient and control groups in terms of the DMFTI (*p* = 0.778). The DI-S, CI-S, OHI-S, GI, and GBTI values, with increased values indicating increased clinical severity, were found to be significantly higher in the patient group than in the control group. Conversely, the MI, PMI, ACR, AFD, CFD, and PFD values, with decreased values indicating an increased degree of resorption and decreased mineral density, were significantly lower in the patient group than in the control group. Regarding the effect size of the results, the DI-S, CI-S, OHI-S, OHI-S, and ACR values had moderate effect sizes, whereas the GI, GBTI, MI, PMI, CFD, AFD, and PFD values had high effect sizes (Table 2).

In the correlation analysis, the OHI-S, DI-S, CI-S, and GI were significantly correlated with the GBTI in the control group (Table 3). In the control group, a moderate correlation was observed between the OHI-S and the GI (Table 3). In the patient group, the GI, OHI-S, DI-S, CI-S, and CI-S were weakly correlated with the GBTI (Table 3). The FB, PMI, MI, and ACR values of all included individuals in the two groups were moderately highly correlated, and these values were statistically significant (Table 4).

Measurement reliability was assessed using the ICC and Spearman’s rho. The ICC, which ranges between 0 and 1, was above 0.84 in all measurements. As a result, it was observed that the measurements showed a high degree of consistency and reliability (Table 5).

**Table 5:** Intraclass correlation coefficient.

	ICC	95% confidence interval	Spearman’s rho
CFD	0.844	0.690–0.924	0.872
AFD	0.948	0.890–0.975	0.956
PFD	0.945	0.886–0.974	0.963
OHI-S	0.982	0.962–0.992	0.947
GI	0.920	0.834–0.962	0.953
GBTI	0.999	0.998–1.000	0.996
ACR	0.949	0.893–0.976	0.950
PMİ	0.957	0.910–0.980	0.955
Mİ	0.978	0.952–0.990	0.971

ICC: intraclass correlation coefficient, CFD: condyle fractal dimension, AFD: angulus fractal dimension, PFD: premolar fractal dimension, OHI-S: simplified oral hygiene index, GI: gingival index, GBTI: gingival bleeding time index, ACR: alveolar crest ratio, PMI: panoramic mandibular index, MI: mental index.

## Discussion

Patients with hemophilia in this study had significantly higher DI-S, CI-S, and OHI-S scores than did those of the control group. Studies conducted in Malaysia, Northern Ireland, and Germany have shown that the oral hygiene status of patients with hemophilia is better than that of the normal population, whereas Czajkowska et al. found no difference between the hemophilia and control groups [17–21]. Differences in health systems, socioeconomic levels, and preventive oral care services may also be contributing factors. In contrast, in studies conducted in Poland and Pakistan, the oral hygiene scores of patients with hemophilia were lower than those of controls. Mielnik-Błaszczak et al. [22] attributed this situation to the fact that the health systems of their countries were not as developed as those in Scandinavian countries, whereas Azhar et al. [23] attributed this to the fact that spontaneous bleeding occurring in patients was a discouraging factor. According to the results of our study, the oral hygiene status of patients with hemophilia in our country was lower than that of the healthy population. This may be because individuals with severe hemophilia are afraid of daily oral hygiene procedures for fear of the risk of bleeding that may be caused by trauma or mucosal damage. In 2018, Kumar et al. [24] compared hemophilia and control groups in terms of OHI-S and fear of dentist/dental treatment. As a result, the OHI-S scores of patients with hemophilia (*p* = 0.030) and fear of dentist/dental treatment (hemophilia = 17%, control = 4%) were found to be higher than those of the control group. This emphasizes the importance of informing patients with hemophilia by determining their OHI-S scores [24]. Patients may also avoid dental treatments because of the need for factor loading before dental treatment, which may cause the progression of the condition [25]. Some studies reported that patients with severe hemophilia who experience bleeding episodes throughout their lives may have impaired somatosensory perception and altered pain perception [26]. This may explain the patients’ fear of daily oral hygiene procedures that may cause pain or the treatment of advanced dental diseases.

To the best of our knowledge, there is no study in the literature on the GBTI of patients with hemophilia; however, the GI and bleeding on probing (BoP) have been examined. Parvaie et al. [18] found no significant difference in the GI between patients with hemophilia and the control group but found that the GI values increased significantly with increasing age. In a study conducted in Türkiye, there were no differences in the GI scores between the patient and control groups. In a study conducted by Czajkowska et al., [21] BoP was found to be significantly lower in the control group than in the patients with hemophilia group.

According to the results of our study, the GI and GBTI scores, which indicate the inflammatory response of the periodontium to poor oral hygiene, were found to be significantly higher in the patient group with hemophilia than in the healthy control group. To evaluate the effect of oral hygiene status on oral bleeding, the correlations among the GI, GBTI, and OHI-S values were analyzed. As expected, these values showed a strong correlation in the control group and a weak correlation in the hemophilic group. Debris or calculus accumulated on the tooth as a result of poor oral hygiene affects the periodontium by causing inflammation, affecting the GI and GBTI. However, because the oral mucosa is affected by hemophilia, and even if the periodontium is healthy, it may tend to bleed even with mild stimulation, which may lead to an increase in GI scores. In particular, when periodontal disease occurs in patients with severe hemophilia, bleeding in the gums may occur even with mild trauma owing to the presence of a high number of enlarged capillaries close to the surface where the gingival epithelium has thinned. Thus, the GI may be high even if the oral hygiene status is good, especially in patients with severe hemophilia. Regarding the oral hygiene status of patients with hemophilia, it should be noted that the GI may not always clearly indicate the presence of inflammation in the periodontium.

In our study, the DMFTI and caries scores of the patient and control groups were similar. This may be because hemophilia does not cause any abnormality in the enamel and dentin structure [25]. In addition, the DMFTI and caries scores were not only dependent on the oral hygiene status but could be significantly influenced by smoking, drug use, and dietary habits. The relatively low mean age (37.56 ± 11.62 years) was also believed to be a reason for the low DMFT rate. These scores may be higher for patient groups with higher mean ages, considering that previous studies have reported that the DMFTI increases with age [27, 28].

A study by Messenger et al. in 2023 showed that the presence of an inhibitor increases the probability of bleeding after tooth extraction. However, none of the individuals included in our study developed inhibitors, and they did not receive prophylactic treatment. Therefore, future studies are needed to include patients with hemophilia who have developed inhibitors or are using prophylaxis [29].

The bone mineral density in patients with hemophilia was first measured by Gallacher et al. [30]; they examined two patients with severe hemophilia, aged 31 and 20 years, who had sustained lumbar bone and femoral neck fractures, respectively. Subsequently, 17 additional patients were included in the study and compared with 19 age- and sex-matched healthy participants. The lumbar bone and femoral neck measurements showed significantly lower bone density in patients with hemophilia. These findings were later confirmed in several studies [5, 31]. In a meta-analysis conducted by Paschou et al. in 2014 [32], the femoral neck and lumbar bones of both pediatric and adult patients were examined. The relationship between severe hemophilia and low bone density was confirmed, and the authors concluded that the decline in bone density started during childhood. Different studies analyzing the mechanisms underlying the potential pathogenesis of factor deficiency in bone metabolism have put forward different views. Some research suggests that factor deficiencies play a role in bone metabolism by directly interfering with the RANK/RANKL/OPG axis and/or the Wnt/β pathway or indirectly by modulating cytokines and/or other factors [7, 33, 34]. Some studies suggest that factor VIII and IX deficiency is associated with inhibition of FX activation and, thus, failure of thrombin generation. Because thrombin can cleave osteopontin, which is important for the binding of osteoclasts to the mineralized matrix, it plays a role in bone remodeling, stimulating osteoblast proliferation, and inhibiting osteoblast differentiation and apoptosis [7].

In the literature, most of the bone mineral density measurements of patients with hemophilia were performed using dual X-ray absorptiometry, and to the best of our knowledge, no study has examined the mineral density of the jaw bones of patients with hemophilia. In a recent study conducted by Czajkowska et al., [10] the PMI and MI were analyzed as radiographic markers of decreased bone mass in patients with hemophilia. Although a significant difference was found in the PMI values in patients with hemophilia, no difference was found in the MI values between the patient and control groups. In our study, the PMI, MI, and ACR were significantly lower in patients with hemophilia than in the controls. Our results support the findings of Czajkowska et al. regarding the importance of PMI as a marker for evaluating factor 8 and 9 deficiencies. The lack of a significant difference in MI values found in the study by Czajkowska et al. may be attributed to the insufficient sample size and variability in disease severity.

FA has been used in many studies to determine postoperative bone changes after oral surgical procedures, changes in bone tissues around implants [35], and changes in the condylar structure [36]. In addition, the effects of metabolic diseases, such as osteoporosis, thalassemia major, and sickle cell anemia, on jaw bones have been studied using FA [37]. In the present study, FD values calculated from the results of the FA of the condyle, angulus, and premolar regions were found to be significantly lower in patients with hemophilia than in the control group. When we observed the correlation between FA, which shows bone density, and MI, PMI, and ACR, which show the amount of resorption, a moderate to high correlation was obtained in 97 patients in whom these values were measured in total. The scales used in this study showed good internal consistency, and the results obtained are similar to those reported in the literature.

The microarchitecture of the trabecular bone was evaluated using FA calculations. The FD values were higher in the regions with complex trabecular structures. The PMI, MI, and ACR values provide information on the cortical bone. Therefore, FA may be useful for treatments in which preserving the trabecular structure of the bone is important, such as intraosseous implant treatment and radiomorphometric analyses, and in cases in which preserving the cortical structure of the bone is important, such as jaw fracture or orthognathic surgical procedures. The low FD values in the condylar region, which is unlikely to be exposed to direct trauma, may be linked to low bone density in patients with hemophilia. The temporomandibular joint region may be sensitive in patients with severe hemophilia, and bruxism should be treated promptly. Occlusal protective splints should be used to protect patients from hemarthrosis and trauma. When planning an interventional surgical procedure in the joint region, bone architecture should be considered, as hemophilia may affect the trabecular bone structure.

The fact that all mandibular bone measurements were low in patients with hemophilia is important for both oral surgeons and hematologists. To minimize or eliminate the need for surgical procedures, even simple ones such as tooth extraction, the importance of oral hygiene should be emphasized, and dental problems should be addressed as noninvasively as possible. If tooth extraction is unavoidable, the condition of the bone should be considered in addition to taking the precautions prescribed by the WHF. Given the high OHI-S scores and GBTI values observed in patients with hemophilia in the study, it is recommended that a thorough assessment of OHI-S scores be performed prior to tooth extraction. When high OHI-S scores are detected, interventions should be considered to improve oral hygiene. Lowering OHI-S scores may reduce edema caused by inadequate oral hygiene and excessive bleeding that may occur after tooth extraction.

In conclusion, we found that the oral hygiene values of patients with hemophilia were lower than those of the normal population. The bone remodeling cycle may be disrupted, and precautions should be taken to prevent bone healing problems after tooth extraction. Clinicians should especially consider potential bone changes when planning to treat these patients. Because bone formation is also affected by tooth extraction, prosthetic rehabilitation should be performed immediately. Patient compliance should be monitored, and surgical procedures should be avoided before implantation or prosthesis placement if there is no change in oral hygiene. Notably, advanced surgical procedures carry a high risk of bleeding. Considering that bone turnover is disrupted in these patients, the risk of bone graft failure may also be high. The risks should be explained to all patients with hemophilia, and the frequency of controls should be increased.

As the jaw bones may show osteoporotic changes, the risk of fracture may be high in the presence of trauma. Additional plate screws for rigid fixation should be considered for treating fractures. If orthognathic surgery is planned, the bone may be insufficient in terms of load-bearing capacity, and additional measures, such as additional plate screws for stabilization or prolongation of the fixation period, should be considered.

Our study had some limitations. It only included individuals with severe hemophilia and thus consisted of a relatively small number of individuals. Moreover, it was conducted on individuals who applied to Hacettepe University in a specific time period and thus has limitations typical of cross-sectional studies. Furthermore, due to the limited number of patients with hemophilia B in our study, it was not feasible to analyze the relationships between hemophilia subgroups.

To enhance our understanding of dental health, the clinical course of the disease, its symptoms, and treatment options for patients with hemophilia, it is recommended that future research be conducted with a more diverse cohort, including a wider age range, a larger number of patients representing various hemophilia subgroups, and the inclusion of female patients, which would provide a more comprehensive perspective on the subject.

## Data Availability

The datasets generated and/or analyzed during the current study are available at https://docs.google.com/spreadsheets/d/1q5VkuTlb8O7uG1p6W_xuqIxjf1kmww7Yb71qoV78hb8/edit?usp=sharing.
